# Editorial: Advances in the understanding of pathogenesis, diagnosis, and therapy of light chain amyloidosis

**DOI:** 10.3389/fonc.2023.1163490

**Published:** 2023-02-24

**Authors:** Divaya Bhutani, Susan Bal, Samuel Rubinstein

**Affiliations:** ^1^ Department of Medicine, Division of Hematology, Columbia University Medical Center, New York, NY, United States; ^2^ Department of Medicine , Division of Hematology, University of Alabama Birmingham, Birmingham, AL, United States; ^3^ Department of Medicine , Division of Hematology, University of North Carolina-Chapel Hill, Chapel Hill, NC, United States

**Keywords:** AL amyloidosis, plasma cell disease, monoclonal gammopathy, B lymphoproliferative disorders, immune complex deposition, urinary extracellular vesicles, localized amyloidosis

Amyloid light chain (AL) amyloidosis is a clonal plasma cell disorder leading to production of misfolded monoclonal immunoglobulin light chains which result in organ damage, most commonly of the cardiac, renal, gastrointestinal, and neurologic systems (1–3). The aim of this series is to collate a series of articles describing advances in the understanding of pathogenesis, diagnosis, and therapy of AL amyloidosis.

Jensen et al. present a review outlining the presenting symptoms and supportive care management of AL amyloidosis with respective organ involvement. The major supportive care challenge in management of patients with AL amyloidosis is management of diastolic heart failure caused by infiltrative cardiomyopathy, which can be further worsened by autonomic neuropathy. Hemodynamics can further be worsened by hypoalbuminemia caused by proteinuria seen with renal involvement. As the authors point out, agents used in management of patients with other etiologies of systolic heart failure such as ACE inhibitors and beta-blockers tend to be poorly tolerated in patients with amyloid cardiomyopathy and should be avoided. Instead, agents to improve the blood pressure such as oral midodrine can be valuable in improving symptoms. Supportive care of patients with cardiac involvement requires judicious management of volume, careful monitoring and management of associated arrhythmias including anticoagulation where indicated. Given challenges in management, multidisciplinary care involving cardiologists, nephrologists, and other specialists with expertise in managing organ damage due to AL amyloidosis is essential.

Cooper et al. present a novel method for assessment of AL amyloidosis using urinary extracellular vesicles, or uEVs. uEVs are nanoparticles secreted by renal epithelial cells and in patients with renal AL amyloidosis, have been shown to contain presence of pathogenic light chain oligomers. Current response assessment in patients with AL amyloidosis is based on measurement of serum light chain levels, but among the patients who achieve a deep response it becomes challenging to identify the presence of residual clonal light chains. The authors have previously described the presence of uEVs containing low-level amyloidogenic light chains as a potential mechanism of ongoing renal injury. The authors find that a minimum of 15 µg of non-albumin uEV protein was the threshold amount needed to detect LC oligomers and based on that developed an equation to estimate the required amount of urine sample for each patient. Furthermore, the authors describe methods to mitigate the issue of immunoglobulin cross-reactivity in the uEV’s to differentiate pathogenic light chain oligomers from other immunoglobulins. Overall, this approach represents a potential non-invasive method to assess hematologic response in patients with renal AL amyloidosis and offers the potential to further explore the pathogenesis of organ progression in these patients.

Patients newly diagnosed with AL amyloidosis can have evidence of alternate mechanisms of paraprotein-mediated organ damage. Yan et al. present novel data describing the outcomes of patients with AL amyloidosis presenting with concomitant renal immune complex (IC) deposition. They analyzed the outcomes of 73 patients proven to have AL amyloidosis by renal biopsy, 19 of whom had concomitant renal IC deposition. Compared to patients with AL amyloidosis and no IC deposition, patients with renal IC deposition have lower rate of hematologic response, a trend towards lower renal survival, and reduced overall survival. These results suggest that renal IC deposition may be a harbinger of an underlying plasma cell clone that is more resistant to the plasma cell-directed therapies that were used: either bortezomib and dexamethasone, melphalan and dexamethasone, or cyclophosphamide, thalidomide, and dexamethasone. Earlier introduction of novel agents, such as daratumumab in settings where it is available, should be considered in patients presenting with concomitant AL amyloidosis and renal IC deposition.

While most cases of AL amyloidosis are driven by an underlying plasma cell neoplasm, occasionally B cell non-Hodgkin lymphoma (NHL) may produce amyloidogenic light chain. When caused by NHL [most frequently, lymphoplasmacytic lymphoma/Waldenstrom’s macroglobulinemia (LPL/WM)], IgM paraprotein is often produced. Berkowitz et al. summarize IgM related amyloidosis and highlight its unique diagnostic and management considerations. Compared to non-IgM AL amyloidosis, IgM related amyloidosis often causes pulmonary, peripheral nerve and soft tissue involvement with cardiac involvement being less common. Infrequent cardiac involvement translates into lower Mayo stage disease at time of diagnosis in a higher proportion of patients. However, response to therapy and outcomes of IgM related amyloidosis compared to non-IgM amyloidosis when matched for stage remain inferior. Management recommendations are based on small studies and expert opinion and predominantly involve targeting the underlying NHL clone using rituximab-based regimens and first and second generation Bruton’s tyrosine kinase inhibitors. Given its distinct clinical presentation and management, presence of an IgM paraprotein must elicit work up for both amyloidosis and underlying NHL.

Martinez et al. conclude the series with a review on the management of AL amyloidosis presenting without systemic involvement, also known as localized AL amyloidosis, which most commongly effects the skin, upper airway, and gastrointestinal tract. Although the prevailing view is that localized AL amyloidosis arises from production of amyloidogenic light chain by tissue-intrinsic plasma cells and lymphocytes, the cellular and molecular pathogenesis of this condition is not well-understood. Patients presenting with apparently localized AL amyloidosis should undergo a thorough evaluation for systemic disease, however in the vast majority of cases, no systemic disease is identified, and patients are at low risk to develop systemic disease in the future. At most, therapy to ameliorate symptoms caused by localized AL amyloid deposition is indicated, and patients typically have an excellent prognosis.

## Author contributions

All authors listed have made a substantial, direct, and intellectual contribution to the work and approved it for publication.

## References

[1] Merlini G Wechalekar AD Palladini G. Systemic light chain amyloidosis: An update for treating physicians. Blood (2013) 121(26):5124–30. doi: 10.1182/blood-2013-01-453001 23670179

[2] Palladini G Hegenbart U Milani P. A staging system for renal outcome and early markers of renal response to chemotherapy in AL amyloidosis. Blood (2014) 124(15):2325–32. doi: 10.1182/blood-2014-04-570010 25115890

[3] Dispenzieri A Gertz MA Kyle RA Lacy M. Q Burritt M. F. Therneau T. M. Serum cardiac troponins and n-terminal pro-brain natriuretic peptide: a staging system for primary systemic amyloidosis. J Clin Oncol (2004) 22(18):3751–7. doi: 10.1200/JCO.2004.03.029 15365071

